# Characterization of the Effects of Cross-Linking of Macrophage CD44 Associated with Increased Phagocytosis of Apoptotic PMN

**DOI:** 10.1371/journal.pone.0033142

**Published:** 2012-03-09

**Authors:** Simon P. Hart, Adriano G. Rossi, Christopher Haslett, Ian Dransfield

**Affiliations:** 1 Division of Cardiovascular and Respiratory Studies, Hull York Medical School/University of Hull, Castle Hill Hospital, Hull, United Kingdom; 2 MRC and University of Edinburgh Centre for Inflammation Research, Queen's Medical Research Institute, University of Edinburgh, Edinburgh, United Kingdom; Instituto de Biofisica Carlos Chagas Filho, Universidade Federal do Rio de Janeiro, Brazil

## Abstract

Control of macrophage capacity for apoptotic cell clearance by soluble mediators such as cytokines, prostaglandins and lipoxins, serum proteins, and glucocorticoids may critically determine the rate at which inflammation resolves. Previous studies suggested that macrophage capacity for clearance of apoptotic neutrophils was profoundly altered following binding of CD44 antibodies. We have used a number of different approaches to further define the mechanism by which CD44 rapidly and specifically augment phagocytosis of apoptotic neutrophils. Use of Fab' fragments unequivocally demonstrated a requirement for cross-linking of macrophage surface CD44. The molecular mechanism of CD44-augmented phagocytosis was shown to be opsonin-independent and to be distinct from the Mer/protein S pathway induced by glucocorticoids and was not functional for clearance of apoptotic eosinophils. CD44-cross-linking also altered macrophage migration and induced cytoskeletal re-organisation together with phosphorylation of paxillin and activation of Rac2. Investigation of signal transduction pathways that might be critical for CD44 augmentation of phagocytosis revealed that Ca^2+^ signalling, PI-3 kinase pathways and altered cAMP signalling were not involved, but did implicate a key role for tyrosine phosphorylation events. Finally, although CD44 antibodies were able to augment phagocytosis of apoptotic neutrophils by murine peritoneal and bone marrow-derived macrophages, we did not observe a difference in the clearance of neutrophils following induction of peritonitis with thioglycollate in CD44-deficient animals. Together, these data demonstrate that CD44 cross-linking induces a serum opsonin-independent mechanism of macrophage phagocytosis of apoptotic neutrophils that is associated with reduced macrophage migration and cytoskeletal reorganisation.

## Introduction

Development of novel, effective therapeutic strategies for treatment of inflammatory diseases requires an understanding the cellular and molecular mechanisms underlying development and progression of inflammation [1]. In particular, neutrophil granulocytes are recruited in large numbers in response to infection or tissue injury and although they represent a vital component of the body's response to infectious agents, release of their formidable array of toxic substances may inflict damage on surrounding tissue and propagate the inflammatory response [2]. Neutrophil-driven inflammation and tissue injury is thought to be a key pathological process in many diseases including rheumatoid arthritis [3], pulmonary fibrosis [4], the adult respiratory distress syndrome [5], and inflammatory bowel disease [6] that are characterized by a failure in the process of resolution of inflammation, resulting in progression to chronic inflammation and scarring [7].

A critical event in the resolution of inflammatory responses is the clearance of recruited inflammatory granulocytes, particularly via the co-ordinated induction of programmed cell death (apoptosis) and subsequent clearance of apoptotic cells by tissue phagocytes [8]. This mechanism has been elegantly confirmed in experimental models of inflammation, where acceleration of neutrophil apoptosis facilitates early resolution and reduction in tissue injury [9]. Neutrophil apoptosis results in loss of expression and function of adhesion molecules [10] and greatly reduced responsiveness to external stimuli [11], leading to functional isolation from micro-environmental stimuli. In addition, apoptotic neutrophils are swiftly recognised and ingested by neighbouring phagocytes, thereby limiting release of harmful intracellular contents [12]. Although multiple molecular mechanisms may be involved in the clearance of apoptotic cells by phagocytes [13], uptake of apoptotic cells suppresses toll-like receptor-driven production of pro-inflammatory mediators by macrophages and can induce release of IL-10 and TGF-β that have the potential to exert anti-inflammatory effects [14], [15].

There is now compelling evidence that defective clearance of apoptotic cells can profoundly influence development of inflammatory disease [16], [17] and autoimmunity [18]. Thus, regulation of macrophage capacity for apoptotic cell clearance by production and release of soluble mediators such as cytokines [19], prostaglandins and lipoxins [20], [21], serum proteins [22], and glucocorticoid hormones [23] may critically determine inflammatory resolution and suppression of autoimmune responses. Our previous work implicated the multifunctional cell surface receptor CD44 as a key regulator of macrophage capacity for phagocytosis of apoptotic cells [24]. The CD44 gene can undergo a complex pattern of alternative splicing, resulting in the expression of different protein isoforms that exhibit distinct functional attributes [25]. CD44 is a receptor for hylauronan [25] and potentially a number of other ligands including E-selectin [26]. Cell surface CD44 acts to control assembly of signalling “platforms” that can regulate cellular behaviour including migration, proliferation and differentiation [27].

We demonstrated that human macrophage phagocytosis of apoptotic PMN was rapidly and specifically augmented (∼1.5 fold increase in the percentage of macrophages capable of phagocytosis of apoptotic PMN and with multiple internalised apoptotic PMN per macrophage equating to a 4-fold increase in phagocytic index) following pre-incubation with CD44 monoclonal antibodies. Although we used microscopy of trypsinised macrophages to confirm that augmented phagocytosis was specific for apoptotic PMN, the underlying mechanism was not determined [24]. In this manuscript, we use a number of different approaches to further define the mechanism by which CD44 antibodies act to rapidly and specifically augment phagocytosis of apoptotic neutrophils.

## Materials and Methods

### Antibodies and other reagents

Reagents were obtained from Sigma-Aldrich (www.sigma-aldrich.com) unless otherwise stated. Iscove's DMEM (IDMEM) was from Invitrogen (www.invitrogen.com). Dextran and Percoll™ were from GE Healthcare (www.gehealthcare.com). Dexamethasone was obtained from David Bull Laboratories (www.maynepharma.com). Human Protein S was obtained from Enzyme Research (www.enzymeresearch.co.uk). Primary monoclonal antibodies (mAb) were from the following sources: paxillin (IgG1), and anti-phosphotyrosine (PY20, IgG2b) were from Transduction Laboratories (www.bdeurope.com). Rat monoclonal recognising mouse granulocytes (IgG2b, Gr1 - Ly6G) was obtained from R and D systems (www.rndsystems.com). Goat polyclonal antibody against Rac1 and Rac2 was obtained from Pierce (www.pierce-antibodies.com). MAb specific for CD44 and variant isoforms were obtained through the Leukocyte Differentiation Antigen workshop as follows: CD44v3 (clone 3G5, IgG2b), CD44v4 (clone FW11-10-3, IgG2a), CD44v4/5 (clone 3D2, IgG1), CD44v5 (clone VFF-8, IgG1), CD44v6 (clone VFF-18, IgG1), CD44v7 (clone VFF-9, IgG1), CD44v9 (clone FW11-24-17, IgG1). Anti-Mer mAb (IgG1) was from R and D systems. Control mouse immunoglobulins (IgG1 and IgG2a), rat immunoglobulins (IgG2b), rabbit immunoglobulins, and F(ab′)_2_ goat anti-mouse immunoglobulin FITC and HRP conjugates were from DAKO (www.dako.com). The 5A4 and 8D2 mAb (IgG1, human CD44) were generously provided by Dr. Graeme Dougherty (University of Arizona, Tucson, AZ), 10.1 mAb (IgG1, human CD64) was a gift of Prof. Nancy Hogg (Cancer Research UK, London), IM7.8.1 mAb (rat IgG2b, murine CD44) [28] and F4/80 (rat IgG2b, mouse macrophage) were obtained from American Type Culture Collection (www.lgcstandards.com). Hybridomas were maintained in Dulbecco's Modified Eagle's Media containing 10% Foetal calf serum and antibodies purified as described [24].

### Human leukocyte Isolation and culture

Human peripheral blood was obtained from the antecubital vein of healthy volunteers after obtaining informed written consent. Ethics approval for granulocyte isolation was obtained from the Lothian Research Ethics Committee; approval numbers #08/S1103/38 or #1702/95/4/72, at the University of Edinburgh, Queen's Medical Research Institute, where participants were recruited and experimentation was carried out. Blood was collected into tubes containing sodium citrate (1% final concentration) to prevent coagulation. Mononuclear (MNC) and polymorphonuclear (PMN) leukocytes were isolated as described [29]. In brief, erythrocytes were sedimented with 0.6% (w/v) dextran T500 followed by fractionation of leukocytes on a discontinuous Percoll™ gradient (prepared in Ca^2+^/Mg^2+^-free phosphate buffered saline (PBS) with final concentrations of Percoll of 50, 63, and 73%) at 720 g for 20 min. MNC were aspirated from the 50/63 interface, and PMN from the 63/73% interface, and washed three times in PBS (without Ca^2+^/Mg^2+^) before culture. PMN (resuspended at 4×10^6^ cells/ml in IDMEM containing 10% autologous serum) were cultured at 37°C in a 5% CO_2_ atmosphere for 20 h in Falcon tissue culture flasks (www.bdbiosciences.com). Cultured PMN populations were >60% apoptotic as determined by morphological analysis and annexin V binding, and <5% propidium iodide positive. MNC were plated at 4×10^6^ cells/ml in IDMEM and incubated for 45–60 min, at 37°C, 5% CO_2_ after which non adherent lymphocytes removed by washing with HBSS (without Ca^2+^/Mg^2+^). Alternatively, monocytes were isolated by negative selection using monocyte isolation kit II as specified by the manufacturer (www.miltenyibiotech.com) and plated at 0.75×10^6^ cells/ml. Adherent monocytes were then cultured *in vitro* to generate monocyte-derived macrophages for a period of 5–7 days in IDMEM containing 10% autologous serum, with or without the addition of 250 nM dexamethasone as detailed in the figure legends.

### Mice and induction of peritonitis

All mice, housed either in the University of Edinburgh or National Kanker Instituut (Amsterdam) animal facilities were humanely maintained and handled in accordance with the UK Home Office Animals Scientific Procedures Act (Licence Number 60/3829). This licence was approved by the University of Edinburgh Ethical Review Committee (approval ID PL08-08). Mice were used between 8 and 12 weeks of age. C57BL/6J mice were obtained from B&K (B&K, www.bku.com). CD44 −/− animals backcrossed onto a C57BL/6J background were obtained from Amgen (generously provided by Prof. Tak Mak). Lack of CD44 expression in various cell populations was confirmed by PCR and flow cytometry. Tiam-1 −/− mice and strain matched FVB mice were provided by Prof. John Collard and maintained at the Nederlands Kanker Instituut in Amsterdam as described [30]. Sterile peritoneal inflammation was elicited by instillation of 2 ml of 4% Brewer's thioglycollate as described [31]. Elicited cells were harvested at days 1–5 by peritoneal lavage following sacrifice by cervical dislocation, with 2×5 ml of sterile PBS. Cell recovery was estimated by haemocytometer counts and the numbers of macrophages, granulocytes and lymphocytes were estimated from relative percentages of cells by microscopic examination of cytocentrifuge preparations together with flow cytometric analysis using laser scatter characteristics combined with mAb staining.

### Isolation of bone marrow macrophages

Macrophages were prepared from the femurs of mice as described [32]. Briefly the expanded ends of cleaned femurs were removed with a scalpel and the bone marrow was extruded into a sterile tube with 5 ml of DMEM containing antibiotics and 10% FCS down the central cavity of the bone using a syringe with a 19G needle. A single cell suspension was obtained after repeated aspiration and cells were resuspended at 1×10^5^ non-erythrocyte cells/ml in complete DMEM containing 25% FCS and 25% L929-conditioned media as a source of macrophage colony stimulating factor. The media was replaced at 3 and 5 days and bone-marrow derived macrophages were used between day 6–9 following isolation.

### Macrophage phagocytosis assay

Monocyte-derived macrophages were cultured in 48 well tissue culture plates as described above for assessment of phagocytosis of apoptotic cells [33]. For experiments using inhibitors of phagocytosis, monocyte-macrophages were washed once then incubated with phagocytosis inhibitors (at the concentrations described in figure legends) for 15 min prior to the phagocytosis assay. The macrophage monolayer was then overlaid with apoptotic PMN (washed and resuspended at a final concentration of 4×10^6^ cells/ml in IDMEM) and incubated at 37°C, 5% CO_2_ for 30 min. Non ingested PMN were removed by washing in IDMEM and monolayers were then fixed in 2.5% glutaraldehyde. The percentage of macrophages that were positive for staining for myeloperoxidase activity with 0.1 mg/ml dimethoxybenzidine and 0.03% (v/v) hydrogen peroxide was quantified microscopically by counting at least 500 cells in randomly selected fields per well and an average between the duplicate wells calculated.

For flow cytometric determination of phagocytosis [34], PMN were resuspended at 10^7^ cells/ml in IMDM and incubated with 20 µM (final concentration) 5-chloromethylfluorescein diacetate (CMFDA, Life Technologies; www.invitrogen.com) for 20 min and then washed prior to culture for 20 h in IMDM containing autologous serum as described above. Macrophage phagocytosis was assessed by overlaying macrophage monolayers with apoptotic PMN (washed and resuspended at a final concentration of 4×10^6^ cells/ml in IDMEM) and incubated at 37°C, 5% CO_2_ for 30 min. Non-ingested PMN were carefully aspirated off and macrophages detached by addition of trypsin/EDTA. The percentage of macrophages that were fluorescent was then determined by flow cytometric analysis.

For experiments to assess clearance of apoptotic cells *in vivo*, CMFDA-labelled apoptotic human PMN were transferred into the peritoneal cavity of mice [35]. Briefly, 100 µg of CD44 mAb 8D2 or 100 µg of IgG1 control (stock solution at 1 mg/ml in PBS) was injected into the peritoneal cavity of either C57BL/6J wild-type or CD44 −/− mice. After 15 min, 1.5×10^7^ human PMN that had been labelled with CMFDA as described above were injected in 150 µl of PBS into the peritoneal cavity. After a further 7 min, the animals were sacrificed and the peritoneal cavity was lavaged with 5 ml of PBS. Cell recovery was estimated by haemocytometer counts and phagocytosis of PMN was estimated by labelling macrophages with PE-conjugated F4/80 and determining the proportion of dual fluorescent macrophages by flow cytometry.

### Flow cytometry

Flow cytometry for analysis of antibody binding was performed essentially as described [29] with all incubations carried out on ice to prevent internalisation of bound antibody. Macrophages were detached from tissue culture plastic by incubation in PBS containing 2 mM EDTA and 0.5% serum for 15–30 min. After washing with ice-cold PBS containing 0.2% (w/v) bovine serum albumin and 0.1% (w/v) sodium azide (PBN), cells (10^5^/assay) were pre-incubated for 10 min with 20% (v/v) normal rabbit serum to block non specific binding of antibodies to Fcγ receptors. Cells were then incubated with saturating concentrations of mAb for 30 min and washed twice in PBN prior to incubation with FITC-conjugated F(ab′)_2_ goat anti-mouse immunoglobulin (DAKO) for 30 min and washed twice more before analysis using a FACScaliber flow cytometer (www.bdbiosciences.com) with post-acquisition data analysis either using Cellquest (www.bdbiosciences.com), Flowjo (www.flowjo.com) or Weasel (www.wehi.edu.au) software.

### Immunoprecipitation and western blotting

Adherent macrophage cultures were washed with PBS containing0.1 mM NaVO_3_ plus protease inhibitor cocktail (www.roche-applied-science.com), and were lysed by incubation with PBS containing 1% NP-40, 0.1 mM NaVO_3_, and protease inhibitor cocktail, 10 min on ice. Membrane and nuclear material were removed by centrifugation at 14,000 g, 4°C, 30 min. Lysates were “pre-cleared” by incubation with protein-A agarose-coupled rabbit anti-mouse IgG, 4°C, 30 min. The resulting lysates were tested for protein concentration using a detergent compatible protein estimation kit (www.piercenet.com) and equilibrated to contain equivalent levels of protein. 100 µl of lysate (100–150 µg total protein) was incubated with 1 µg of either mouse IgG control or anti-paxillin mAb, 4°C, 30 min, shaking. Immunoprecipitation was achieved by incubation for 30 min with protein-A coupled rabbit anti-mouse IgG and washed twice in Tris buffered saline containing 0.1% Triton X-100, and once in 25 mM Tris plus 0.05% SDS. Samples were resolved using a 9% reducing polyacrylamide gel and transferred electrophoretically (50 V for 1 h) onto nitrocellulose (GE Healthcare). For detection of phosphotyrosine, membranes were blocked with TBS plus 0.05% Tween-20 (TBS-T) all other blots with TBS-T plus 10% non-fat dried milk powder (w/v). Bound antibodies were visualised with enhanced chemiluminescence as described by the manufacturer (Pierce).

### Assay for detection of activated Rac

Adherent macrophage cultures were lysed in RIPA buffer (Sigma) containing protease inhibitor cocktail (Roche) plus 1 mM PMSF. Lysates were cleared of membrane and nuclear material by centrifugation, total protein estimated and levels equilibrated as described for immunoprecipitation. 20 µl of lysate was removed for estimation of total Rac protein and the remaining (approximately 300 µg) was incubated with GST-PAK (CRIB) fusion protein coupled to Sepharose beads, 4°C, 1 h, with constant agitation [23]. Beads were washed 4 times in ice cold Tris buffer (50 mM Tris pH7.2, 150 mM NaCl, 10 mM MgCl_2_, 1% Triton X-100, protease inhibitor cocktail, 1 mM PMSF), and the amount of active Rac1 and Rac2 bound to PAK CRIB domain quantified by SDS PAGE and western blotting as described for immunoprecipitation.

### Statistics

Results are presented as mean ± SEM and n = number of independent experiments using cells obtained from different donors or mice. Differences were analysed by ANOVA either using Mann-Whitney test for comparison of two data sets or the Tukey multiple comparison test using Instat software (www.graphpad.com).

## Results and Discussion

### Analysis of the mechanism of CD44 antibody-mediated augmentation of phagocytosis

**Figure 1:**
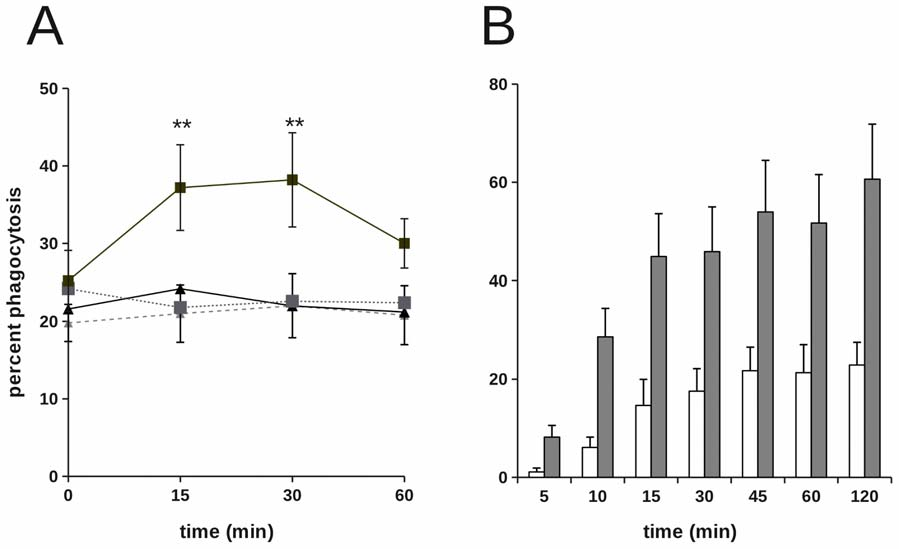
CD44 cross-linking promotes human macrophage phagocytosis of apoptotic human PMN. A) Monocyte-derived macrophages were incubated with CD44 mAb 5A4 Fab′ fragments (squares) or control Fab′ (triangles) for 30 min prior to addition of F(ab′)_2_ goat anti-mouse immunoglobulins (black symbols, solid line) or PBS control (gray symbols, dashed lines). Macrophages were then co-incubated with apoptotic targets at 37°C for the times indicated prior to assessment of phagocytosis by microscopy. Results shown are mean ± SEM for n = 5 separate experiments. ** indicates significant difference between cross-linking versus non-cross-linked (p<0.01). B) Monocyte-derived macrophages were incubated with CD44 mAb for 30 min to allow saturation of binding prior to addition of CMFDA-labelled apoptotic targets at 37°C for the times indicated. Assessment of phagocytosis was made by flow cytometry (white bars = untreated; gray bars (CD44-treated). Data shown are mean percentage phagocytosis ± SEM for 3 independent experiments. At all time points examined except 5 min, CD44-treated cells exhibit significant augmentation of phagocytosis (p<0.05).

We previously reported that F(ab′)_2_ fragments of CD44 mAb were able to augment macrophage phagocytosis of apoptotic cells [24]. These data implicate a requirement for cross-linking of CD44 for the augmentation of phagocytosis, whilst eliminating the possibility that antibody-mediated bridging via FcγR was involved. To examine the requirement for antibody-induced redistribution of cell surface CD44, we pre-treated macrophages with Fab′ fragments of a well characterised CD44 mAb (5A4) [24]. Fab′ fragments of 5A4 showed saturable binding to macrophages in flow cytometry, demonstrating that binding was not compromised by enzymatic digestion used during fragment preparation (data not shown). In the absence of cross-linking, binding of 5A4 mAb Fab′ fragments failed to augment phagocytosis of apoptotic cells (Fig. 1A). When bound 5A4 Fab′ fragments were cross-linked by addition of F(ab′)_2_ anti-mouse immunoglobulins, we observed a rapid increased in the proportion of macrophages capable of phagocytosis of apoptotic PMN, unequivocally demonstrating a requirement for cross-linking of CD44. These data provide important evidence that augmentation of phagocytosis that we observe does not involve antibody blockade of CD44 binding to ligand or “masking” of CD44 epitopes that normally suppress macrophage phagocytosis.

Comparison of phagocytosis by untreated and CD44-treated macrophages in time course experiments indicated that CD44 cross-linking allows the recruitment of previously unresponsive cells and does not simply act to improve the efficiency of phagocytosis in responsive macrophages (Fig. 1B). Interestingly, in these experiments there is an apparent difference in the duration of the effects of CD44 mAb (compare Fig. 1A and 1B). One possibility is that this reflects a difference in the extent of cross-linking achieved using Fab′ and F(ab′)_2_ anti–mouse immunoglobulin (Fig. 1A) when compared with intact antibody (Fig. 1B), However, the rapid effect of cross-linking strongly suggests that CD44 cross-linking initiates intracellular signal transduction events that regulate macrophage phagocytic function.

**Figure 2:**
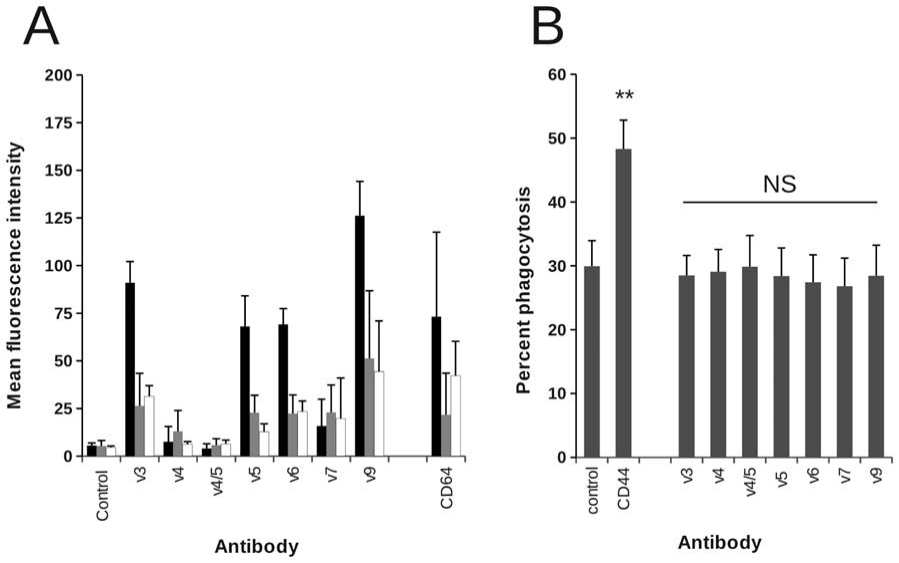
CD44 variant antibodies do not promote phagocytosis of apoptotic PMN. A) Monocytes (white bars) or monocyte-derived macrophages cultured for 5 days in the absence (black bars) or presence of dexamethasone (gray bars) were incubated with different isoform-specific CD44 mAb or CD64 mAb (10.1) for 30 min to allow saturation of binding prior to addition of FITC-conjugated F(ab′)_2_ goat anti-mouse immunoglobulins and assessment of binding by flow cytometry. Data shown are the average mean fluorescence intensity ± SEM for 4 independent experiments. B) Monocyte-derived macrophages were incubated with isoform-specific CD44 mAb for 30 min to allow saturation of binding prior to addition CMFDA-labelled apoptotic targets at 37°C for the times indicated. Assessment of phagocytosis was made by flow cytometry. Data shown are mean percent phagocytosis ± SEM from 4 different macrophage preparations, no significant augmentation of phagocytosis compared with control was observed with any of the variant antibodies examined (NS).

We next examined the binding and functional effects of a large panel of CD44 antibodies including mAb specific for isoforms expressing different alternatively spliced exons. Expression of CD44 variant isoforms was higher on monocyte-derived macrophages (Fig. 2A - black bars) than either glucocorticoid-treated monocyte-derived macrophages (grey bars) or monocytes. In these experiments, we included CD64 (FcγRI) as a myeloid-specific receptor, demonstrating similar patterns of expression to CD44v3, CD44v5, and CD44v6, raising the possibility that this pattern of expression simply reflected differentiation status. However, expression of the haemopoeitic (unspliced) form of CD44 was considerably higher on monocytes than either monocyte-derived macrophage population [mean fluorescence for monocytes = 2037±147; monocyte-derived macrophages = 1471±530; and glucocorticoid-treated macrophages 811±355]. These data would be consistent with induction of expression of variant isoforms during monocyte differentiation and suppression of variant isoform expression by glucocorticoids. Interestingly, all CD44 mAb that bind the haematopoietic form that we have tested potentiate phagocytosis of apoptotic neutrophils ([24] and data not shown), whereas none of the isoform-specific CD44 antibodies had any effect (Fig. 2B). One possibility is that a critical level of cross-linking of membrane CD44 is required to confer a pro-phagocytic signal that is not achieved following binding of the CD44 isoform-specific antibodies. This suggestion would be consistent with data from antibody titration studies which reveal a close correlation between the level of binding of CD44 mAb to the macrophage surface in flow cytometric analysis and augmentation of phagocytosis of apoptotic cells (data not shown). Alternatively, binding of the variant CD44 mAb may not allow the correct juxtaposition of CD44 in the membrane to confer signal transduction events.

### Cell type specificity and role of opsonins in CD44 augmentation of phagocytosis

Our previously published work demonstrated a specific augmentation of phagocytosis of apoptotic human PMN following binding of CD44 mAb to huma monocyte/macrophages, with no effect upon phagocytosis of apoptotic lymphocytes [24]. To further investigate cell-type specificity of the augmentation of phagocytosis following CD44 pre-treatment, we examined the effects of CD44 mAb upon macrophage phagocytosis of apoptotic eosinophils, a cell type that is closely related to the neutrophil in terms of ontogeny and cell surface receptor profile. Although macrophage phagocytosis of apoptotic PMN was augmented following pre-treatment with CD44 mAb (24.6±6.1% phagocytosis for untreated v 45.5±14.2% for CD44-treated), phagocytosis of apoptotic eosinophils was not affected (40±14% phagocytosis for untreated v 38±12.9% for CD44-treated, n = 3). We therefore conclude that the augmentation of phagocytosis of apoptotic PMN is specific for neutrophils.

**Figure 3:**
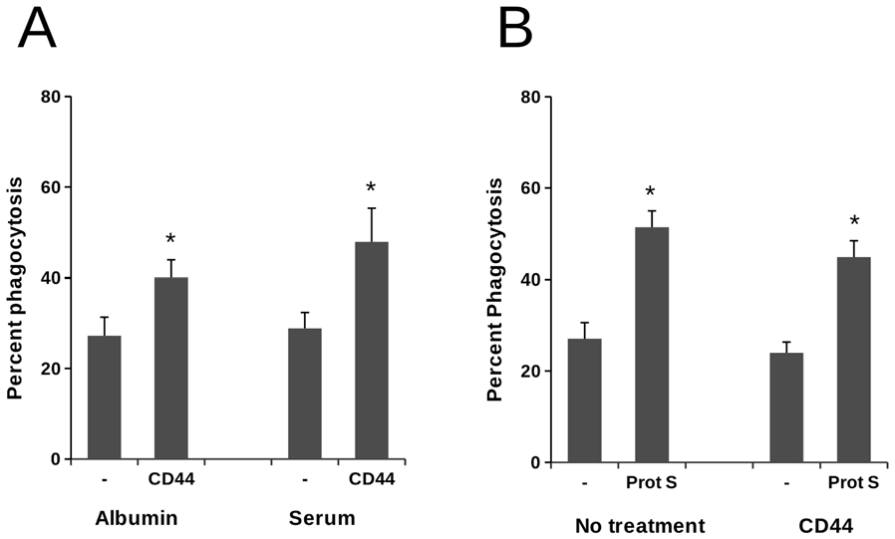
CD44 promotes a serum-opsonin independent mechanism of phagocytosis of apoptotic neutrophils. A) Monocyte-derived macrophages were incubated in the absence or presence of CD44 mAb for 30 min prior to addition CMFDA-labelled neutrophils that had been cultured either in human serum albumin (Albumin) or autologous serum (Serum). Assessment of phagocytosis was made by flow cytometry, data are mean ± SEM from 5 independent experiments. Although CD44 augmented phagocytosis relative to untreated cells (* = p<0.05), no significant differences between phagocytosis of PMN cultured in albumin versus serum was found following CD44 treatment. B) Glucocorticoid-treated monocyte-derived macrophages were incubated in the absence or presence of CD44 mAb for 30 min prior to addition CMFDA-labelled neutrophils that had been cultured in human serum albumin. Phagocytosis was assessed in the presence of 100 ng/ml of protein S for 30 min and the proportion of phagocytic macrophages determined by flow cytometry. Data shown are mean percentage phagocytosis ± SEM from 5 independent experiments. Although the presence of protein S augmented phagocytosis by glucocorticoid-treated macrophages (* = p<0.05), there was no significant difference between phagocytosis of PMN in the presence or absence of protein S following CD44 treatment.

We next examined the requirement for opsonins that are present within serum for the CD44 antibody-mediated augmentation of phagocytosis of apoptotic neutrophils. Experiments using neutrophils that had undergone apoptosis in the presence of human serum albumin alone or in the presence of either heat-inactivated or untreated human serum revealed that CD44 was able to induce macrophage phagocytosis in the absence of opsonins that might be present in serum, including complement components and the phosphatidylserine binding protein S (Fig. 3A). In the presence of serum albumin alone, phagocytosis was increased approximately 1.5-fold from 27.2±9% to 40.1±9%. In the presence of autologous serum, CD44 augmented recognition by a similar percentage, from 28.8±8% to 47.8±16%, an effect that was maintained when neutrophils were cultured in the presence of heat-inactivated serum (data not shown).

The lack of requirement for a serum factor in the augmentation of phagocytosis by CD44 mAb indicates that the mechanism of action is distinct from the augmentation observed following treatment of monocyte-macrophages with glucocorticoids. We therefore investigated whether there were synergistic effects of CD44 cross-linking upon augmentation of phagocytosis of apoptotic cells following treatment with glucocorticoids. Interestingly, these experiments revealed that there was no effect of CD44 mAb upon phagocytosis of apoptotic neutrophils by glucocorticoid-treated macrophages, either in the presence or absence of protein S (Fig. 3B), despite high levels of cell surface expression of CD44 on these cells ([21] and data not shown). Together, these data indicate that CD44 promotes an opsonin-independent pathway for phagocytosis of apoptotic neutrophils that does not involve the Mer/Protein S-dependent mechanism utilised by glucocorticoid-treated macrophages [36].

### CD44-mediated regulation of macrophage cytoskeleton

**Figure 4:**
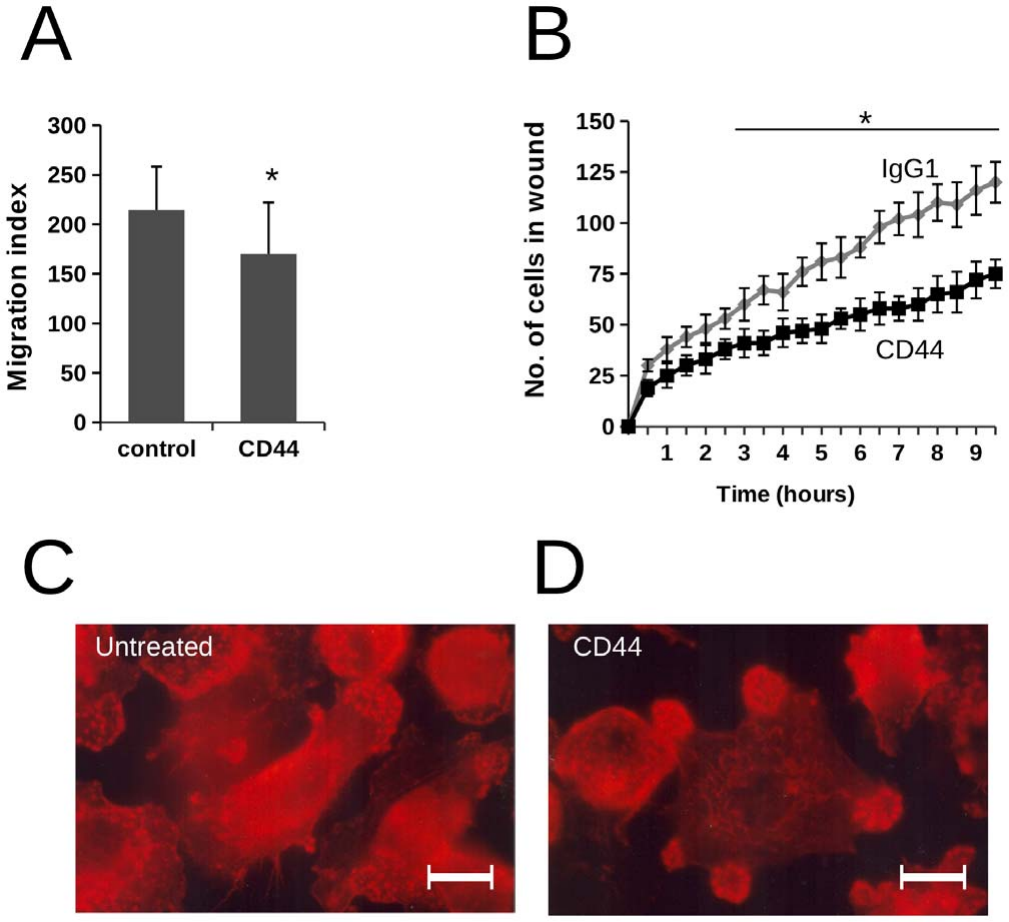
Effect of CD44 on macrophage migration. A) Monocyte-derived macrophages were incubated in the absence or presence of CD44 mAb for 30 min to binding prior to “wounding” of the cell monolayer. Assessment of migration into the wounded area was made by microscopy at 18 h, data shown are mean number of cells present in the wound ± SEM from 6 separate experiments. In paired analysis there is a significant reduction of migration in the presence of CD44 mAb (p<0.05). B) Monocyte-derived macrophages were incubated in the absence or presence of CD44 mAb for 30 min to binding prior to “wounding” of the cell monolayer. Assessment of macrophage migration into the wounded area was made by quantification of the numbers of macrophages in the wound using time lapse microscopy images captured over 10 hours. Measurements for untreated macrophages are indicated by gray diamond symbols and CD44-treated macrophages by black squares. Data are mean number of cells observed in the wound ± SEM from 3 independent experiments. * indicates that migration was significantly reduced by CD44 mAb from 3 h onwards (p<0.05). Monocyte-derived macrophages adherent to glass coverslips were treated with media (C) or CD44 mAb (D) for 30 min prior to fixation with paraformaldehyde and staining for filamentous actin with rhodamine phalloidin. Representative micrographs show localisation of podosome-like structures in adherent macrophages (C) and marked redistribution that is observed following CD44 antibody binding (D). Scale bar = 10 µm.

In view of previous reports demonstrating antibody-induced shedding of CD44 from the plasma membrane [37], we examined whether macrophages treated with CD44 mAb exhibited down-regulation of CD44 expression. However, in a series of experiments where macrophages were pre-treated with CD44 mAb and then cultured at 37°C for up to 3 h, we did not observe reduction in the levels of CD44 expressed at the cell surface by flow cytometry (data not shown). In microscopy analysis, the appearance of macrophages treated with CD44 mAb was suggestive of altered adhesion status. We therefore investigated macrophage capacity for migration following treatment with CD44 mAb using a “scratch” assay in which monolayers of macrophages were disrupted with a pipette tip [38] and the migration of cells monitored using time lapse video microscopy over an 8 hour period. In these assays, the rate of macrophage migration into the wound was significantly attenuated following pre-incubation with CD44 mAb, reducing the rate of macrophage migration by approximately 30% (Fig. 4A and B).

Examination of actin localisation in CD44-treated macrophages using immunofluorescence microscopy revealed rapid alteration of cytoskeletal organisation independently of the presence of apoptotic targets. In the representative micrographs shown in Fig. 4C and D, marked changes in the organisation of podosome-rich regions were apparent following CD44 antibody treatment. Quantification of the numbers of macrophages displaying altered cytoskeletal organisation (29.5±4% untreated macrophages compared with 54.1±4% CD44-treated macrophages showing the presence of multiple patches of podosome-rich regions, n = 3) suggested a role for CD44 cross-linking in the control of cytoskeletal dynamics.

### CD44-mediated macrophage signal transduction events

**Figure 5:**
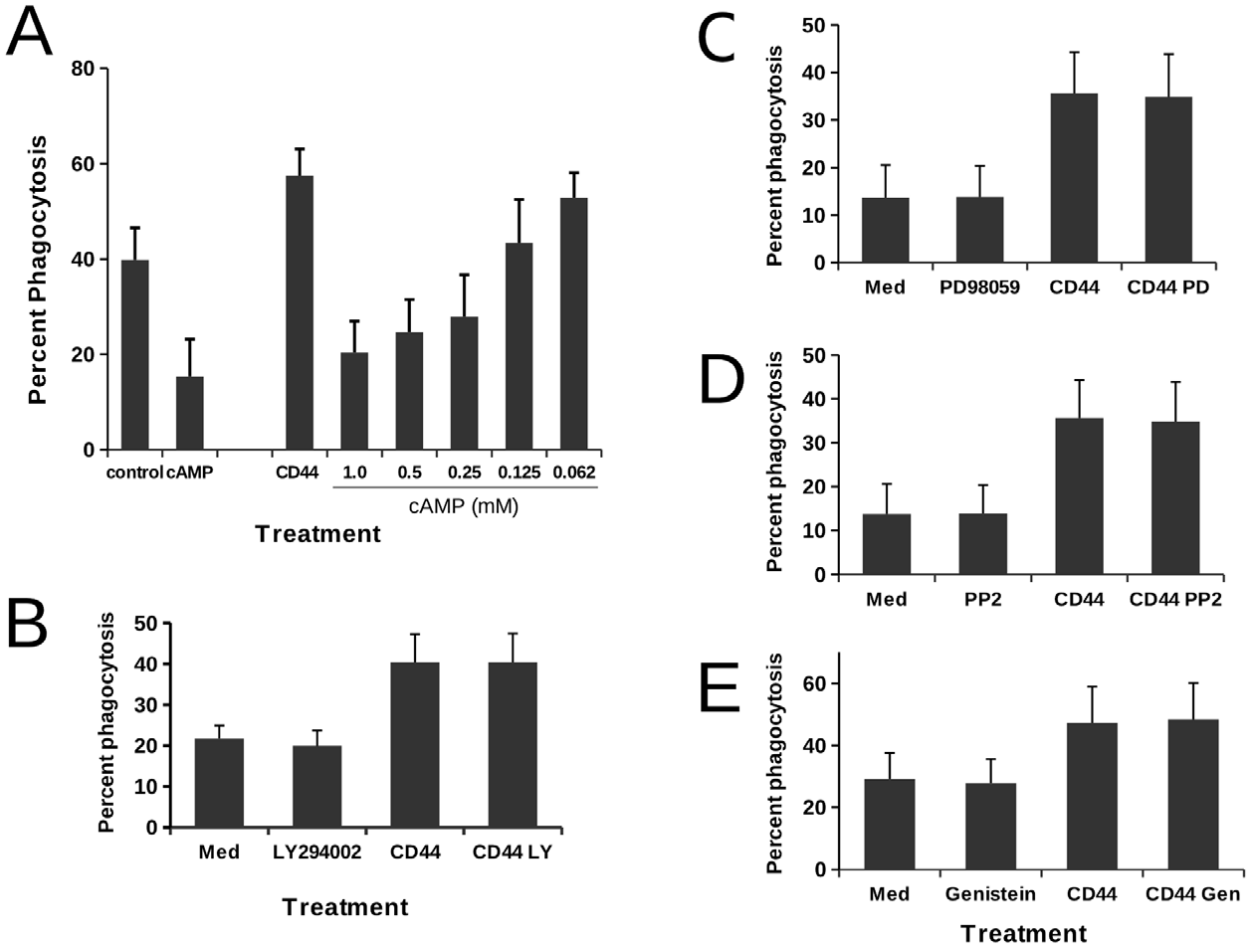
Effects of pharmacological inhibition of signalling pathways in macrophages upon CD44-augmentation of phagocytosis. A) Monocyte-derived macrophages were incubated with CD44 mAb for 30 min prior to addition of dibutyryl cAMP at concentrations shown. Macrophages were then co-incubated with apoptotic targets at 37°C for 30 min prior to assessment of phagocytosis by microscopy. Results shown are mean percentage phagocytosis for 3 independent experiments. B) Monocyte-derived macrophages were pre-incubated with 10 µM LY294002 at 37°C and then co-incubated with CD44 mAb for 30 min prior to addition of CMFDA-labelled apoptotic targets. After 30 min, assessment of phagocytosis was made by flow cytometry. Results shown are mean percentage phagocytosis ± SEM for 5 independent experiments. C)Monocyte-derived macrophages were pre-incubated with 50 nM PD98059 at 37°C and then co-incubated with CD44 mAb for 30 min prior to addition of CMFDA-labelled apoptotic targets. After 30 min, assessment of phagocytosis was made by flow cytometry. Results shown are mean percentage phagocytosis ± SEM for 3 independent experiments. D) Monocyte-derived macrophages were pre-incubated with 25 µM PP2 at 37°C and then co-incubated with CD44 mAb for 30 min prior to addition of CMFDA-labelled apoptotic targets. After 30 min, assessment of phagocytosis was made by flow cytometry. Results shown are mean percentage phagocytosis ± SEM for 4 independent experiments. E) Monocyte-derived macrophages were pre-incubated with 25 µM Genistein at 37°C and then co-incubated with CD44 mAb for 30 min prior to addition of CMFDA-labelled apoptotic targets. After 30 min, assessment of phagocytosis was made by flow cytometry. Results shown are mean percentage phagocytosis ± SEM for 3 independent experiments. In panels B-E there was no statistical difference between the percentage phagocytosis recorded in the presence or absence of pharmacological inhibitor following CD44 mAb treatment.

In terms of specificity of CD44 effects, a number of molecules that might mediate rapid signalling events have been proposed to interact with CD44, particularly with respect to cytoskeletal organisation, including src family kinases (e.g. p56lck) [39] and members of the ezrin/radixin/moesin family [40]. Although we have previously shown that elevation of cAMP within macrophages acts to inhibit phagocytosis, cross-linking of CD44 might lead to altered phagocytosis via redistribution or local alteration of intracellular cAMP levels mediated by CD44 interactions with ezrin, a protein which can serve to anchor protein kinase A [41]. Although the overall levels of phagocytosis were different in untreated and CD44-treated cells, dibutyryl cAMP displayed similar inhibitory effects on phagocytosis (Fig. 5A) with a concentration response that paralleled that previously seen for untreated macrophage phagocytosis of apoptotic neutrophils [41]. We further tested whether inhibition of protein kinase A would be influenced by H-89, a broad-specificity inhibitor of protein kinase A. In these experiments, pretreatment of macrophages with 10 µM H-89 did not affect CD44-mediated augmentation of phagocytosis (Control = 22.6±2.8; H-89 20.2±4.2; CD44-treated = 40.7±2.9; H-89+CD44-treated = 41.9±3.9, n = 4). Our interpretation is that CD44 does not act to augment phagocytosis through effects upon protein kinase A activity.

Since intracellular calcium has a key role in cytoskeletal regulation, we next investigated the impact of binding of CD44 antibodies upon mobilisation of intracellular calcium stores in macrophages. In experiments in which Fura-2 loaded macrophages were treated with CD44 antibodies we did not observe any alteration in baseline levels of intracellular calcium (data not shown), suggesting that calcium release is not required for the effects of CD44 upon either cytoskeletal alteration or functional effects upon phagocytosis or migration. Interestingly, although PI-3K activity has been suggested to be critical for phagocytosis of apoptotic cells [42], we found that blockade of PI3-K with LY 294002 did not affect phagocytic uptake of apoptotic cells by CD44-treated macrophages, despite fully blocking phosphorylation of Akt (Fig. 5B and data not shown).

**Figure 6:**
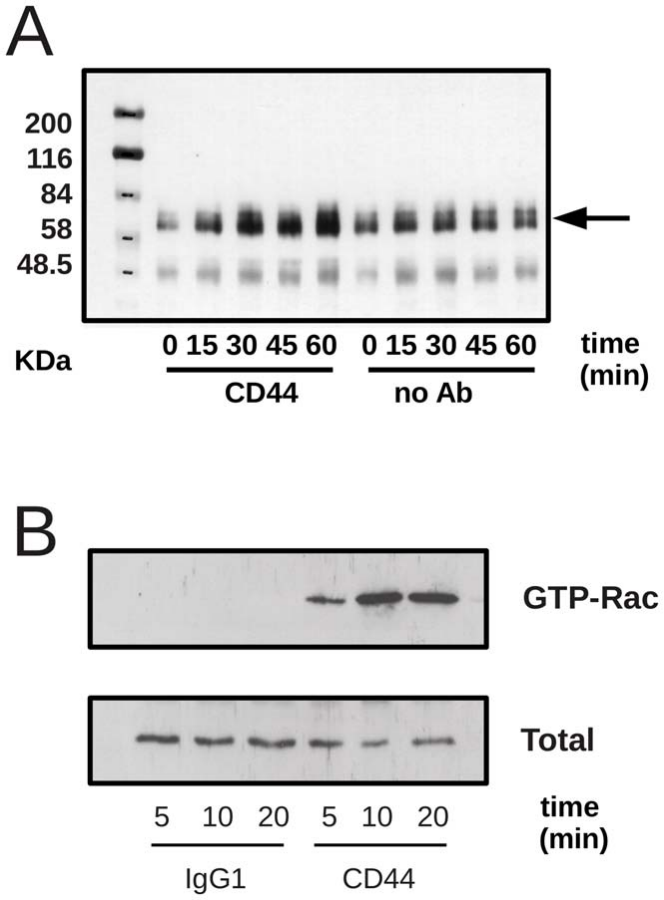
CD44-augmentation of phagocytosis is associated with phosphorylation of paxillin and Rac2 activation. A) Monocyte-derived macrophages were incubated with CD44 mAb at 37°C for different time points up to 60 min prior to lysis in RIPA buffer. Immunoblot analysis of tyrosine phosphorylation of paxillin revealed a time-dependent increase in phosphorylation following CD44 antibody binding. Image shows representative autoradiograph of a single experiment from 3 that were performed. Densitometric analysis of the phosphopaxillin using ImageJ (http://rsbweb.nih.gov/ij/) confirms increased phosphorylation of paxillin following CD44 treatment. For control phosphopaxillin levels at 0, 15, 30, 45 and 60 min was 7.7, 9.9, 9.3 and 8.2 respectively. In contrast, phosphopaxillin levels following CD44 cross-linking were 3.7, 8.8, 13.7, 12.4 and 15.6 at 0, 15, 30, 45 and 60 min respectively. Molecular weight standards shown in KDa. B) Monocyte-derived macrophages were incubated with either control IgG1 or CD44 mAb at 37°C for different times up to 20 min prior to lysis in RIPA buffer as indicated. Pull down assays using PAK CRIB agarose beads revealed a robust increase in GTP-bound Rac2 in the presence but not absence of CD44 antibody binding. A representative autoradiograph from a single experiment (from 5 that were performed) is shown.

We next used a series of inhibitors of key signalling pathways in macrophages, including the broad spectrum tyrosine kinase inhibitor (genistein), the src kinase inhibitor (PP2) and an inhibitor of MEK (PD98059). Surprisingly, we did not observe inhibition of CD44-augmented phagocytosis with any of these inhibitors (Fig. 5C–E), despite their key role in adhesion regulation. However, immunoblot analysis of lysates prepared from CD44-treated macrophages in the absence of apoptotic targets demonstrated that paxillin phosphorylation was increased in a time-dependent manner following CD44 cross-linking (Fig. 6A), consistent with the changes in actin/paxillin-rich podosome structures we observed in CD44-treated macrophages. Although these data implicate tyrosine phosphorylation as an important event in the regulation of macrophage adhesion by CD44, the inhibitor data presented here suggests that tyrosine phosphorylation is not required for the observed augmentation of phagocytosis of apoptotic neutrophils.

The activity of Rho family GTPases (including, Rac and cdc42) has been closely linked to control of apoptotic cell phagocytosis [43]. We utilized “pull-down” assays with the CRIB domain of p21activated kinase to assess the effects of CD44 upon Rac activation. In untreated cells, there was a low basal level of both Rac1 and Rac2 activity. However, following CD44 antibody treatment, we found a rapid and specific induction of Rac2 activation but no effect on Rac1 (Fig. 6B and data not shown). The specificity for CD44 induction of Rac2 activity may explain our observations that CD44 does not augment phagocytosis by non-professional phagocytes such as fibroblasts which do not express Rac2.

### The effects of CD44 mAb on phagocytosis in Tiam-1- and CD44-deficient mice

**Figure 7:**
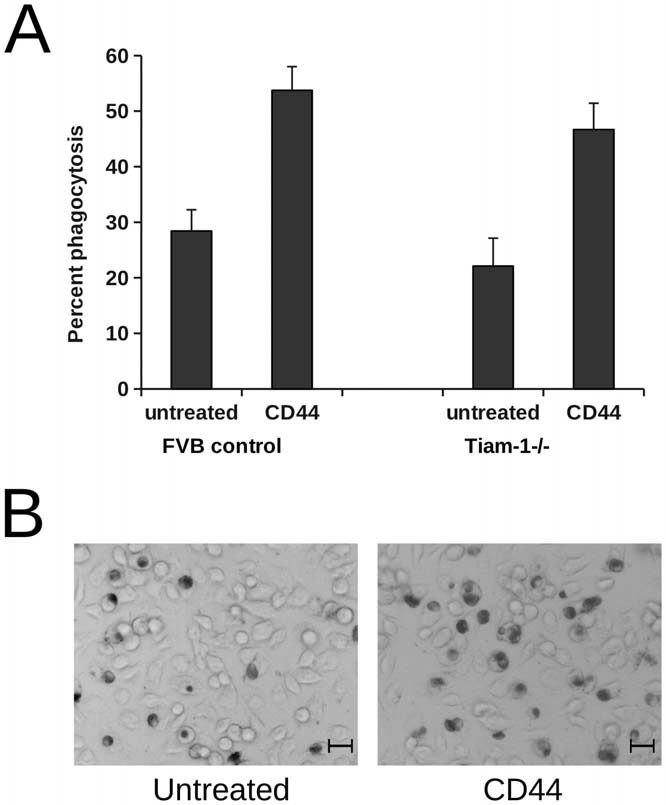
Augmentation of phagocytosis by CD44 in Tiam-1 −/− mice. A) Peritoneal macrophages from wild-type (FVB) and Tiam-1 −/− mice were pre-treated with CD44 mAb IM7.8.1 or rat IgG2b control prior to incubation with apoptotic human PMN. After 30 min phagocytosis of apoptotic PMN was determined by microscopy. Results show mean percentage phagocytosis ± SEM for macrophages derived from 4 separate animals. B) Representative microscopy images showing Tiam1 −/− peritoneal macrophages that have been pre-treated with or without CD44 mAb IM7.8.1 prior to incubation with apoptotic human PMN and then stained for myeloperoxidase activity. Scale bar = 20 µm.

In view of the CD44-induced Rac activation and previous reports of association of CD44 with Tiam-1 [44], we next examined whether macrophages derived from Tiam-1 −/− mice were able to exhibit augmentation of phagocytosis of apoptotic cells following CD44 mAb treatment. Experiments using either peritoneal or bone-marrow-derived macrophages from C57BL/6J wild-type and CD44 −/− mice demonstrated that anti-mouse CD44 mAb IM7 failed to augment phagocytosis of apoptotic human neutrophils in the absence of CD44 expression (wild-type macrophages: 26.9±12.3%; CD44-treated wild-type macrophages: 39.4±15.2%; CD44−/−: 27.1±8.8%; CD44-treated CD44−/−: 29.5±10.8%; n = 6). As shown in Fig. 7A and B, phagocytosis of apoptotic human neutrophils by mouse peritoneal macrophages was augmented following pre-treatment with rat anti-mouse CD44 mAb IM7.8.1. Macrophages from Tiam-1 −/− mice also exhibited increased phagocytosis of apoptotic neutrophils following treatment with CD44 mAb suggesting that Tiam-1 is not required to confer augmented phagocytosis (Fig. 7A and B).

**Figure 8:**
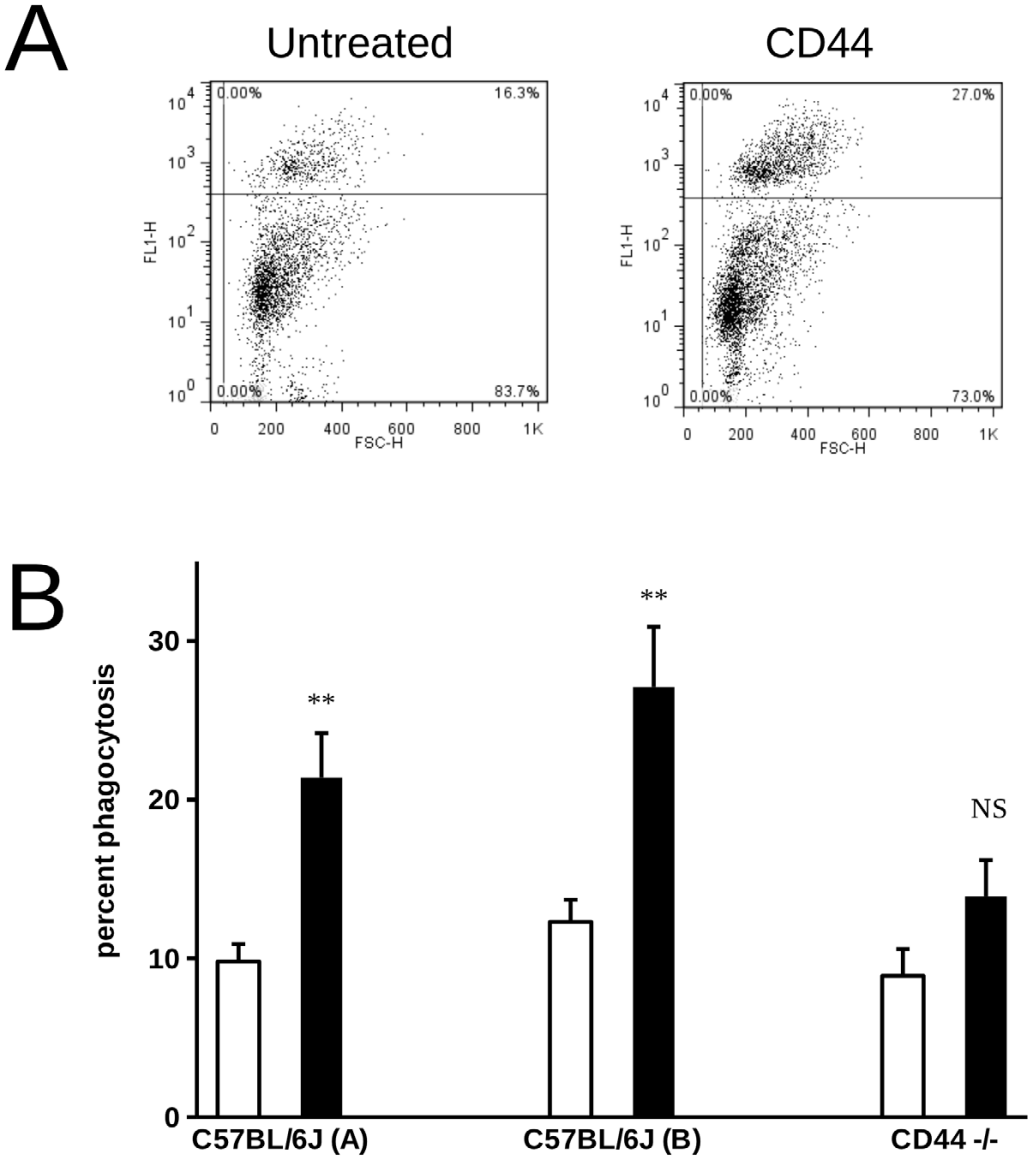
Effects of CD44 cross-linking on phagocytosis of human apoptotic PMN transfered into the peritoneal cavity of mice. A) CMFDA-labelled human apoptotic neutrophils were transferred into the peritoneal cavity of mice that had been previously injected with CD44 mAb, 8D2 or mouse IgG1 control antibody. Phagocytosis of apoptotic cells was determined by flow cytometry following labelling of mouse macrophages with PE-conjugated F4/80. Representative histograms show Forward Scatter versus FL-1 for IgG1 treated versus 8D2 treated animals – representative of 4 independent experiments that were performed. B) Wild-type (C57BL/6J) or CD44−/− mice were pre-injected with either IgG1 (white bars) or CD44 mAb (8D2 – black bars) into the peritoneal cavity prior to injection of human apoptotic neutrophils. Quantification of phagocytosis by peritoneal macrophages lavaged from either wild type (C57BL/6J) or CD44 −/− was made by flow cytometry as described for (A) above. For C57BL/6J (A) results shown are the mean ± SEM for 10 separate animals. For C57BL/6J (B) and CD44 −/−, results are from 4 independent experiments. ** indicates results are statistically significant (p<0.01).

We next used a model of inflammation in which apoptotic human neutrophils were injected into the peritoneal cavity of mice. We pre-injected either a CD44 mAb which cross-reacts with mouse CD44 (clone 8D2) or an IgG1 control into the peritoneal cavity prior to adoptive transfer of neutrophils. The proportion of macrophages which phagocytosed apoptotic neutrophils was then estimated by flow cytometry as described [35]. Consistent with published data [35], results from preliminary experiments indicated that clearance of apoptotic cells occurred extremely rapidly and we therefore used an time point of 7 minutes to examine the earliest stages of apoptotic cell clearance when we would predict CD44 cross-linking to have the greatest impact. In these experiments, CD44 mAb pre-treatment resulted in augmentation of phagocytosis (Fig. 8A and B), suggesting that CD44 cross-linking was capable of augmentation of apoptotic cell phagocytosis *in vivo*. Importantly, in experiments using CD44 −/− mice, pre-injection of CD44 mAb had no significant effect on macrophage phagocytosis, ruling out the possibility that opsonisation of apoptotic targets accounted for the observed effects (Fig. 8B). We did not examine whether there was augmentation of phagocytosis of apoptotic neutrophils from CD44 −/− by wild type macrophages. Thus, we cannot exclude the interesting possibility that CD44 mAb are able to potentiate phagocytosis by bridging apoptotic neutrophils, but not apoptotic eosinophils or apoptotic lymphocytes to the macrophage surface.

Previously published studies reported that CD44 deficiency exacerbates inflammation in the lung, increasing neutrophil recruitment and compromising resolution of inflammation [45], [46]. Similarly, lack of CD44 increased macrophage numbers within atherosclerotic lesions in ApoE deficient animals [47] suggesting that CD44 acts as a critical regulator of inflammatory cell migration *in vivo*. However, inflammatory cell recruitment in the peritoneal cavity is not affected by lack of CD44 as inflammatory cell numbers in response to LPS or *E. coli* infection in the peritoneal cavity is similar in wild-type and CD44 −/− mice [48]. Since our data using adoptive transfer of apoptotic human neutrophils suggests that CD44 ligation can augment phagocytic clearance, we sought to examine whether lack of CD44 expression would influence progression and resolution of inflammation in the peritoneal cavity. Our *in vitro* data would predict that lack of CD44 expression may delay clearance of apoptotic neutrophils and possibly promote emigration of macrophages from inflammatory sites. In the absence of CD44 expression, neutrophil clearance might be delayed and macrophage emigration from the peritoneal cavity might be enhanced.

**Figure 9:**
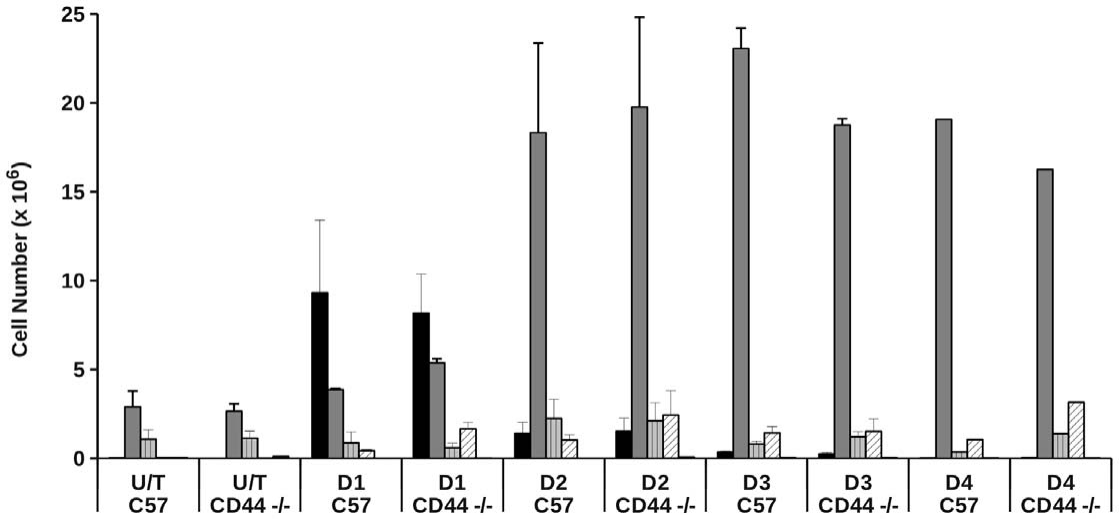
Thioglycollate-induced cell recruitment patterns in CD44 −/− animals. Peritonitis was induced in wild type (C57BL/6J) or CD44−/− animals by injection of 2.5 ml of thioglycollate. At time points indicated, estimates of the total cell counts and percentage of cell types present in the peritoneal lavage fluid was made by microscopy and flow cytometry and the total counts for different cell populations calculated. Data are mean cell numbers ± SEM from 5 separate experimental animals. Black bars: neutrophils, dark grey bars: macrophages, grey hatched bars: lymphocytes, white hatched bars: eosinophils, other bar: mast cells.

We therefore undertook a temporal analysis (days 1–4) of the cellular infiltrates in experimental thioglycollate-induced peritonitis in wild-type and CD44-deficient animals. There was no difference in the cellular composition of lavage obtained from wild-type or CD44-deficient animals in the absence of any treatment (Fig. 9). Following thioglycollate-induced sterile peritonitis, we observed increased numbers of neutrophils in the peritoneal cavity of both wild-type and CD44−/− animals after 24 h. By day 2, neutrophil numbers in the peritoneal lavage were lower in both wild-type and CD44−/− animals, with increased numbers of cells with monocyte/macrophage morphology, consistent with the pattern of cell recruitment we have seen previously following thioglycollate treatment [31]. There was a trend for higher numbers of eosinophils in the lavage of CD44 −/− animals, although this did not reach statistical significance.

Although data presented here suggest that lack of CD44 expression does not affect cell recruitment or clearance, it remains possible that CD44 may be differentially involved in the regulation of inflammatory cell numbers in different anatomic compartments. Consistent with this suggestion, CD44 may play an important role in the recruitment of macrophages to atherosclerotic lesions, possibly by promoting expression of VCAM-1 [47]. Furthermore, although CD44−/− lymphocytes were found to traffic normally following transfer into naïve wild-type animals, clear differences were observed in trafficking of CD44−/− cells in arthritic recipients [49]. These studies raise the possibility that the influence of CD44 on cellular recruitment, clearance and emigration patterns may depend on both the tissue type and the inflammatory stimulus. In addition there may be compensatory mechanisms involved that compensate for the lack of CD44 expression in different tissues/organs.

### Conclusions 

We present evidence that definitively demonstrates cross-linking of macrophage CD44 rapidly and specifically augments macrophage phagocytosis of apoptotic neutrophils. Promotion of the phagocytic phenotype following CD44 cross-linking induces rapid re-organisation of paxillin and actin containing “podosome-like” adhesions, altered phosphorylation of paxillin and activation of Rac2. These cytoskeletal rearrangements are associated with reduced macrophage migration following CD44 cross-linking and we speculate that CD44 cross-linking favours the stabilisation of interaction of macrophages with apoptotic neutrophil targets that promotes their subsequent internalisation.
